# Supplementary material for: Characteristics of Retinitis Pigmentosa Associated with ADGRV1 and Comparison with USH2A in Patients from a Multicentric Usher Syndrome Study Treatrush
Source: Int J Mol Sci. 2021 Sep 26;22(19):10352. doi: 10.3390/ijms221910352 (PMC8509029; doi:10.3390/ijms221910352)
Supplement: Supplementary file 1 [file ijms-22-10352-s001.zip › ijms-1381333-supplementary.pdf]

International Journal of
Molecular Sciences

MDPI

Supplemental Figure S1

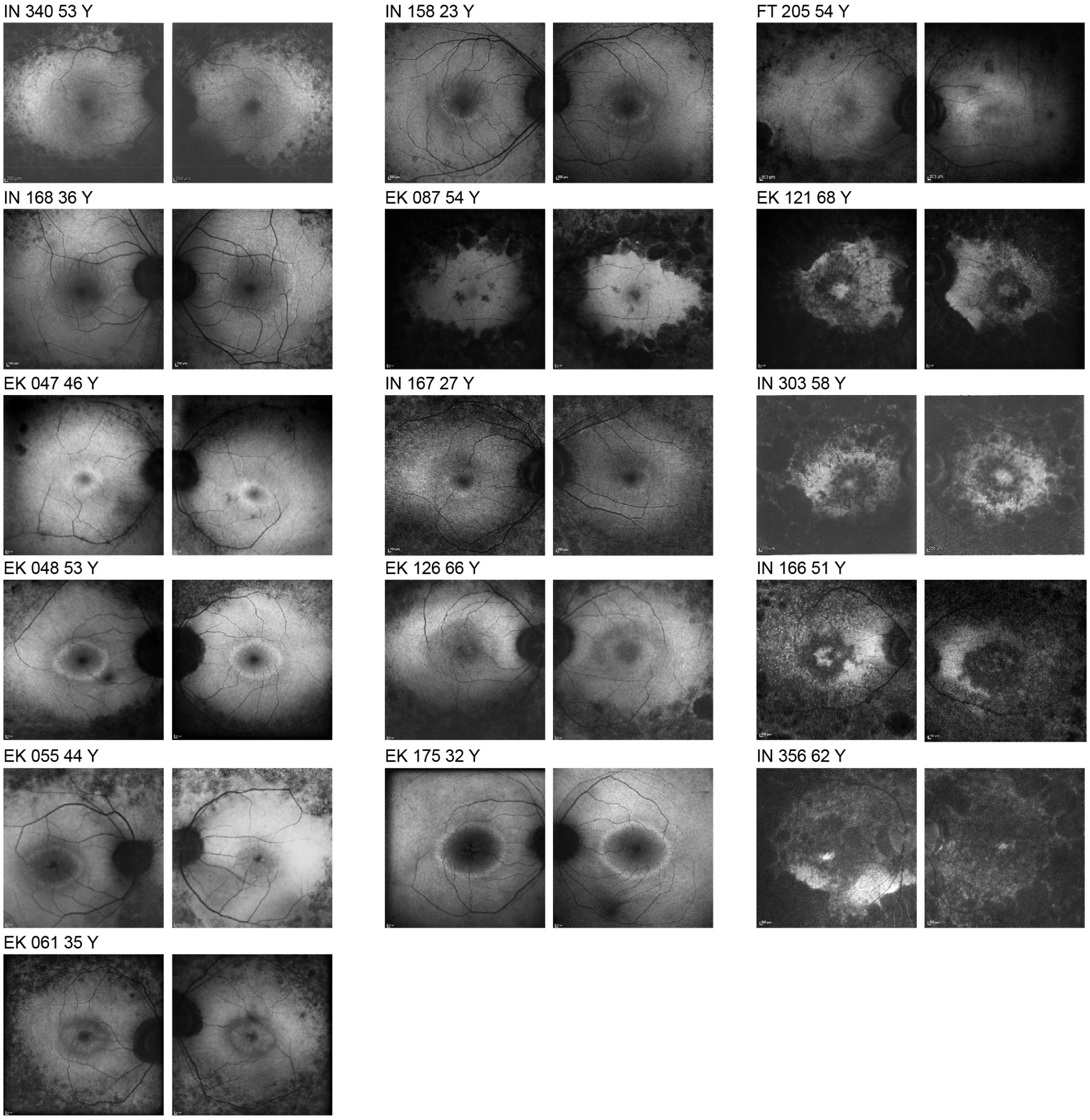

Figure S1. FAF patterns of right and left eyes of *ADGVR1* patients. ID and age of each patient is stated above the two images of each patient (in each column, the right eye is shown on the left and left eye is shown on the right). First two columns show hyperautofluorescent rings with preserved central retina while the third column shows advanced disease - hyperautofluorescent patch and atrophy. Note older ages of patients in the last column. Note also good interocular symmetry.

Supplemental Table S1

Pathogenic variants of studied patients.

Treatrush ID	Reference ID	Reference	CENTRE	GENE	Variant 1	Variant2
TR_EK-009	DE03U06091	Bonnet et al, 2016	GERMANY	*USH2A*	p.(Cys536ter)	p.(Glu767Serfs*21)
TR_EK-010	DE03U07100	Bonnet et al, 2016	GERMANY	*USH2A*	p.(Arg626ter)	p.(Gly516Arg)
TR_EK-012	DE03U13121	Bonnet et al, 2016	GERMANY	*USH2A*	p.(Arg3538ter)	p.(Glu767Serfs*21)
TR_EK-013	DE03U13121	Bonnet et al, 2016	GERMANY	*USH2A*	p.(Arg3538ter)	p.(Glu767Serfs*21)
TR_EK-014	DE03U21141	Bonnet et al, 2016	GERMANY	*USH2A*	p.(Glu767Serfs*21)	p.(Ile3103Val)

TR_EK-016	DE03U25161	Bonnet et al, 2016	GERMANY	*USH2A*	p.(Val218Glu)	p.(Cys419Phe)
TR_EK-029	DE03U26290	Bonnet et al, 2016	GERMANY	*USH2A*	p.(Glu4458Aspfs*3)	p.(Cys4140Phe)
TR_EK-032	DE03U27321+	Bonnet et al, 2016	GERMANY	*USH2A*	p.(=,Tyr318Cysfs*17)	p.(Ile5166Val)
TR_EK-035	DE03U27321+	Bonnet et al, 2016	GERMANY	*USH2A*	p.(=,Tyr318Cysfs*17)	p.(Ile5166Val)
TR_EK-036	DE03U28360	Bonnet et al, 2016	GERMANY	*USH2A*	p.(Leu2380Profs*37)	p.(Gly3647Ser)
TR_EK-038	DE03U29381	Bonnet et al, 2016	GERMANY	*USH2A*	c.5776+1G>A	p.(Ala3944Asp)
TR_EK-039	DE03U30390	Bonnet et al, 2016	GERMANY	*USH2A*	p.(Trp2945ter)	c.4627+25435_4987+660del
TR_EK-040	DE03U31401	Bonnet et al, 2016	GERMANY	*USH2A*	p.(Glu767Serfs*21)	c.(3157+1_3158-1)_(4627+1_4628-1)del
TR_EK-046	DE03U33461	Bonnet et al, 2016	GERMANY	*USH2A*	p.(Trp3955ter)	p.(Ser259Phefs*63)
TR_EK-049	DE03U34490	Bonnet et al, 2016	GERMANY	*USH2A*	p.(Glu767Serfs*21)	c.11549-1G>A
TR_EK-051	DE03U35510	Bonnet et al, 2016	GERMANY	*USH2A*	p.(Gln4711ter)	c.5776+2T>C
TR_EK-053	DE03U36530	Bonnet et al, 2016	GERMANY	*USH2A*	p.(Glu767Serfs*21)	p.(Trp2075Cys)
TR_EK-054	DE03U37541	Bonnet et al, 2016	GERMANY	*USH2A*	p.(Glu767Serfs*21)	p.(Trp3521Arg)
TR_EK-056	DE03U38561	Bonnet et al, 2016	GERMANY	*USH2A*	p.(Glu767Serfs*21)	p.(Cys2309Phe)
TR_EK-058	DE03U02581	Bonnet et al, 2016	GERMANY	*USH2A*	p.(Glu767Serfs*21)	p.(Glu767Serfs*21)
TR_EK-066	DE03U39661	Bonnet et al, 2016	GERMANY	*USH2A*	p.(Cys870ter)	p.(Arg4192Cys)
TR_EK-067	DE03U40671	Bonnet et al, 2016	GERMANY	*USH2A*	p.(Glu767Serfs*21)	c.7595-2144A>G
TR_EK-069	DE03U41701+	Bonnet et al, 2016	GERMANY	*USH2A*	p.(Glu767Serfs*21)	c.(11548+1_11549-1)_(11711+1_11712-1)del
TR_EK-070	DE03U41701+	Bonnet et al, 2016	GERMANY	*USH2A*	p.(Glu767Serfs*21)	c.(11548+1_11549-1)_(11711+1_11712-1)del
TR_EK-071	DE03U42710	Bonnet et al, 2016	GERMANY	*USH2A*	p.(Trp3955ter)	p.(=,Tyr318Cysfs*17)
TR_EK-072	N/A	Bonnet et al, 2016	GERMANY	*USH2A*	p.(Trp3955ter)	p.(Glu4445_Ser4449delinsAspLeu)
TR_EK-073	DE03U43730	Bonnet et al, 2016	GERMANY	*USH2A*	c.11549-1G>A	c.(2993+1_2994-1)_(13811+1_13812-1)dup
TR_EK-075	DE03U44750	Bonnet et al, 2016	GERMANY	*USH2A*	p.(Trp3702ter)	p.(Trp3702ter)
TR_EK-077	DE03U45770	Bonnet et al, 2016	GERMANY	*USH2A*	c.5777-2A>G	p.(Trp3521Arg)
TR_EK-080	DE03U03800	Bonnet et al, 2016	GERMANY	*USH2A*	p.(Glu767Serfs*21)	p.(Glu767Serfs*21)
TR_EK-081	DE03U46811	Bonnet et al, 2016	GERMANY	*USH2A*	p.(Trp3955ter)	p.(Asn346His)
TR_EK-082	DE03U47821	Bonnet et al, 2016	GERMANY	*USH2A*	p.(Cys870ter)	p.(Thr2919Pro)
TR_EK-084	DE03U48840	Bonnet et al, 2016	GERMANY	*USH2A*	c.14791+2T>C	c.9259-2402_9371+1537del
TR_EK-085	DE03U49851	Bonnet et al, 2016	GERMANY	*USH2A*	p.(Trp3702ter)	p.(Trp3702ter)
TR_EK-088	N/A	Glocke et al, 2014	GERMANY	*USH2A*	p.(Val4367Argfs*22)	c.8682-1665_8845+140del
TR_EK-093	DE03U04931	Bonnet et al, 2016	GERMANY	*USH2A*	p.(Glu767Serfs*21)	p.(Glu767Serfs*21)
TR_EK-095	DE03U50950	Bonnet et al, 2016	GERMANY	*USH2A*	p.(Asn2651Glnfs*10)	p.(=,Tyr318Cysfs*17)

TR_EK-096	DE03U51960	Bonnet et al, 2016	GERMANY	*USH2A*	p.(Glu767Serfs*21)	p.(Glu4458Aspfs*3)
TR_EK-097	N/A		GERMANY	*USH2A*	c.5776+1G>A	c.5776+1G>A
TR_EK-098	DE03U52980	Bonnet et al, 2016	GERMANY	*USH2A*	p.(Trp3955ter)	p.(Gln4676Profs*7)
TR_EK-099	DE03U53990	Bonnet et al, 2016	GERMANY	*USH2A*	c.785-6636_1840+208del	p.(Trp3521Arg)
TR_EK-100	DE03U081001	Bonnet et al, 2016	GERMANY	*USH2A*	p.(Cys520Arg)	p.(Cys520Arg)
TR_EK-101	DE03U091011	Bonnet et al, 2016	GERMANY	*USH2A*	p.(Glu767Serfs*21)	p.(Trp3521Arg)
TR_EK-102	DE03U101020	Bonnet et al, 2016	GERMANY	*USH2A*	p.(Trp2945ter)	p.(Cys2128Tyr)
TR_EK-104	DE03U111041	Bonnet et al, 2016	GERMANY	*USH2A*	p.(Gln4711ter)	p.(Trp3521Arg)
TR_EK-114	DE03U121140	Bonnet et al, 2016	GERMANY	*USH2A*	p.(Asn4079Trpfs*19)	p.(Asn4079Trpfs*19)
TR_EK-120	DE03U141201	Bonnet et al, 2016	GERMANY	*USH2A*	p.(Arg4935ter)	p.(Gly3142ter)
TR_EK-124	DE03U011240	Bonnet et al, 2016	GERMANY	*USH2A*	p.(Glu767Serfs*21)	p.(Glu767Serfs*21)
TR_EK-125	DE03U151250	Bonnet et al, 2016	GERMANY	*USH2A*	p.(Tyr1103ter)	p.(His340Asp)
TR_EK-127	N/A		GERMANY	*USH2A*	p.(Glu767Serfs*21)	p.(Glu767Serfs*21)
TR_EK-128	DE03U161282	Bonnet et al, 2016	GERMANY	*USH2A*	p.(Gln4235ter)	p.(Glu767Serfs*21)
TR_EK-129	DE03U171292	Bonnet et al, 2016	GERMANY	*USH2A*	p.(Trp3955ter)	c.(11548+1_11549-1)_(11711+1_11712-1)del
TR_EK-130	DE03U181302	Bonnet et al, 2016	GERMANY	*USH2A*	p.(Cys3090ter)	p.(Trp3955ter)
TR_EK-131	DE03U191312	Bonnet et al, 2016	GERMANY	*USH2A*	c.6805+2T>C	p.(Arg334Trp)
TR_EK-134	DE03U201342	Bonnet et al, 2016	GERMANY	*USH2A*	p.(Trp2841ter)	c.9258+1G>A
TR_EK-148	DE03U221482	Bonnet et al, 2016	GERMANY	*USH2A*	p.(Trp3955ter)	c.1144-2A>T
TR_EK-149	DE03U231492	Bonnet et al, 2016	GERMANY	*USH2A*	p.(Glu767Serfs*21)	p.(Val218Glu)
TR_EK-150	DE03U241502	Bonnet et al, 2016	GERMANY	*USH2A*	p.(Trp3955ter)	p.(Val218Glu)
TR_EK-160	N/A		GERMANY	*USH2A*	p.(His308Serfs*16)	p.(Glu767Serfs*21)
TR_EK-179	N/A	Neuhaus et al 2017	GERMANY	*USH2A*	p.(Trp3955ter)	p.(Leu2215Serfs*16)
TR_EK-259	N/A		GERMANY	*USH2A*	p.(Trp2841ter)	p.(Gly3142ter)
TR_EK-278	N/A		GERMANY	*USH2A*	c.7301-1G>A	p.(Glu2238Ala)
TR_EK-279	N/A		GERMANY	*USH2A*	c.(11711+1_11712-1)_(12066+1_12067-1)del	c.(11711+1_11712-1)_(12066+1_12067-1)del
TR_FT-115	IT02U111320	Bonnet et al, 2016	ITALY	*USH2A*	p.(Trp1084ter)	p.(Glu767Serfs*21)
TR_FT-184	IT02U2420650	Bonnet et al, 2016	ITALY	*USH2A*	p.(Phe4993Profs*7)	p.(Phe4993Profs*7)
TR_FT-187	IT02U1916760	Bonnet et al, 2016	ITALY	*USH2A*	p.(Tyr4329ter)	p.(Gly516Val)
TR_FT-189	IT02U1414820	Bonnet et al, 2016	ITALY	*USH2A*	p.(Trp211ter)	p.(Gly4340Arg)
TR_FT-191	IT02U1314641	Bonnet et al, 2016	ITALY	*USH2A*	p.(Glu767Serfs*21)	p.(Pro309Leu)
TR_FT-197	IT02U0610141	Bonnet et al, 2016	ITALY	*USH2A*	c.1841-2A>G	p.(Leu3606Pro)
TR_FT-199	IT02U2521551	Bonnet et al, 2016	ITALY	*USH2A*	p.(Cys1228ter)	p.(Thr3571Met)
TR_FT-200	IT02U337021	Bonnet et al, 2016	ITALY	*USH2A*	p.(Phe1297Serfs*17)	p.(Thr3571Met)
TR_FT-203	IT02U304071	Bonnet et al, 2016	ITALY	*USH2A*	p.(Trp3955ter)	p.(Glu4963Glyfs*38)
TR_FT-211	IT02U2219552	Bonnet et al, 2016	ITALY	*USH2A*	p.(Tyr2566Leufs*11)	p.(Arg3119Cys)
TR_FT-212	IT02U0710160	Bonnet et al, 2016	ITALY	*USH2A*	p.(Gln1573ter)	p.(Thr3571Met)

TR_FT-213	IT02U0410110	Bonnet et al, 2016	ITALY	*USH2A*	p.(Arg737ter)	p.(Ile285Thr)
TR_FT-214	IT02U0310050	Bonnet et al, 2016	ITALY	*USH2A*	c.14791+4A>G	p.(Thr3571Met)
TR_FT-216	IT02U293311	Bonnet et al, 2016	ITALY	*USH2A*	p.(Arg2509Glyfs*19)	p.(Arg1777Trp)
TR_FT-217	IT02U272481	Bonnet et al, 2016	ITALY	*USH2A*	p.(Trp3955ter)	c.(3316+1_3317-1)_(4627+1_4628-1)del
TR_FT-219	IT02U348231	Bonnet et al, 2016	ITALY	*USH2A*	p.(Phe1297Serfs*17)	p.(Thr3571Met)
TR_FT-220	IT02U262321	Bonnet et al, 2016	ITALY	*USH2A*	p.(Ser2639Pro)	p.(Ser2639Pro)
TR_FT-223	IT02U2319930	Bonnet et al, 2016	ITALY	*USH2A*	p.(Glu1492ter)	p.(Val2700Glu)
TR_FT-294	IT02U1816640 +	Bonnet et al, 2016	ITALY	*USH2A*	p.(Ser2969ter)	p.(Ser2969ter)
TR_FT-295	IT02U1816640 +	Bonnet et al, 2016	ITALY	*USH2A*	p.(Ser2969ter)	p.(Ser2969ter)
TR_FT-301	IT02U2017672 +	Bonnet et al, 2016	ITALY	*USH2A*	c.7452-1G>A	c.7452-1G>A
TR_FT-345	IT02U1012180	Bonnet et al, 2016	ITALY	*USH2A*	p.(Thr3571Met)	p.(Thr352Ile)
TR_FT-418	IT02U0110011	Bonnet et al, 2016	ITALY	*USH2A*	p.(Cys953ter)	p.(Trp3955ter)
TR_FT-419	IT02U1615200	Bonnet et al, 2016	ITALY	*USH2A*	p.(Trp3955ter)	p.(Glu767Serfs*21)
TR_FT-426	IT02U0810602	Bonnet et al, 2016	ITALY	*USH2A*	p.(Phe4993Profs*7)	p.(Phe4993Profs*7)
TR_FT-427	IT02U0210041	Bonnet et al, 2016	ITALY	*USH2A*	p.(Asn330Lysfs*8)	p.(Thr3571Met)
TR_FT-428	IT02U1816640 +	Bonnet et al, 2016	ITALY	*USH2A*	p.(Ser2969ter)	p.(Ser2969ter)
TR_FT-429	IT02U1214361	Bonnet et al, 2016	ITALY	*USH2A*	p.(Tyr3715ter)	p.(Thr3571Met)
TR_FT-435	IT02U2118741	Bonnet et al, 2016	ITALY	*USH2A*	p.(Glu2242ter)	p.(Arg303His)
TR_FT-436	IT02U0510130	Bonnet et al, 2016	ITALY	*USH2A*	p.(Ser1350Leufs*16)	p.(Gly268Arg)
TR_FT-438	IT02U31410	Bonnet et al, 2016	ITALY	*USH2A*	p.(Thr3571Met)	p.(Thr3571Met)
TR_FT-439	IT02U091070	Bonnet et al, 2016	ITALY	*USH2A*	p.(Glu767Serfs*21)	p.(Pro2241Thr)
TR_FT-442	IT02U282841	Bonnet et al, 2016	ITALY	*USH2A*	p.(Thr3571Met)	p.(Thr3571Met)
TR_FT-443	IT02U1515091	Bonnet et al, 2016	ITALY	*USH2A*	p.(Phe4993Profs*7)	p.(Tyr90Cys)
TR_FT-445	IT02U1715361	Bonnet et al, 2016	ITALY	*USH2A*	p.(Gln4750ter)	p.(Met3271Cysfs*30)
TR_FT-449	IT02U2017672 +	Bonnet et al, 2016	ITALY	*USH2A*	c.7452-1G>A	c.7452-1G>A
TR_FT-451	IT02U32601	Bonnet et al, 2016	ITALY	*USH2A*	p.(Phe1297Serfs*17)	c.(4251+1_4252-1)_(4396+1_4397-1)del
TR_IN-024	U6	Bonnet et al, 2011	FRANCE	*USH2A*	p.(Glu3562ter)	p.(Glu767Serfs*21)
TR_IN-025	U21	Bonnet et al, 2011	FRANCE	*USH2A*	c.10586-1G>C	p.(Tyr1730Trpfs*6)
TR_IN-026	U24	Bonnet et al, 2011	FRANCE	*USH2A*	p.(Ser1307ter)	p.(Cys536Arg)
TR_IN-027	U30	Bonnet et al, 2011	FRANCE	*USH2A*	p.(Glu767Serfs*21)	p.(Arg303His)
TR_IN-028	FR02U06392+	Bonnet et al, 2016	FRANCE	*USH2A*	p.(Glu767Serfs*21)	p.(Tyr4128Hisfs*24)
TR_IN-159	FR02U113302	Bonnet et al, 2011	FRANCE	*USH2A*	p.(Tyr4031ter)	p.(Phe4697Leufs*2)
TR_IN-161	FR02U06392+	Bonnet et al, 2016	FRANCE	*USH2A*	p.(Glu767Serfs*21)	p.(Tyr4128Hisfs*24)
TR_IN-162	FR02U102062	Bonnet et al, 2016	FRANCE	*USH2A*	p.(His308Serfs*16)	c.(1644+1_1645-1)_(4627+1_4628-1)del

TR_IN-163	FR02U091752	Bonnet et al, 2016	FRANCE	*USH2A*	p.(Arg4115Cys)/p.(Thr4425Met)	p.(Thr4439Ile)
TR_IN-164	FR02U08872	Bonnet et al, 2016	FRANCE	*USH2A*	c.12067-2A>G	c.12067-2A>G
TR_IN-176	FR02U4660482	Bonnet et al, 2016	FRANCE	*USH2A*	c.7595-2144A>G	c.7595-3Cys>G
TR_IN-177	FR02U4256782	Bonnet et al, 2016	FRANCE	*USH2A*	p.(Arg34ter)	p.(Arg3719His)
TR_IN-180	FR02U4559922	Bonnet et al, 2016	FRANCE	*USH2A*	p.(Glu767Serfs*21)	p.(Val218Glu)
TR_IN-185	FR02U2225512	Bonnet et al, 2016	FRANCE	*USH2A*	p.(Glu287ter)	p.(Asn346His)
TR_IN-186	FR02U146642	Bonnet et al, 2016	FRANCE	*USH2A*	p.(Gln919ter)	p.(Glu767Serfs*21)
TR_IN-209	FR02U123922	Bonnet et al, 2016	FRANCE	*USH2A*	p.(Arg4935ter)	p.(His308Serfs*16)
TR_IN-238	FR02U07472+	Bonnet et al, 2016	FRANCE	*USH2A*	p.(Trp3955ter)	p.(Thr1443Pro)
TR_IN-239	FR02U07472+	Bonnet et al, 2016	FRANCE	*USH2A*	p.(Trp3955ter)	p.(Thr1443Pro)
TR_IN-243	FR02U1610392+	Bonnet et al, 2016	FRANCE	*USH2A*	p.(Glu284Aspfs*38)	c.7595-2144A>G
TR_IN-258	FR02U159252	Bonnet et al, 2016	FRANCE	*USH2A*	p.(=,Tyr318Cysfs*17)	p.(Arg998Lys)
TR_IN-262	FR02U2529982	Bonnet et al, 2016	FRANCE	*USH2A*	p.(Pro5127Argfs*8)	p.(Pro5127Argfs*8)
TR_IN-264	FR02U1610392+	Bonnet et al, 2016	FRANCE	*USH2A*	p.(Glu284Aspfs*38)	c.7595-2144A>G
TR_IN-268	FR02U2119962	Bonnet et al, 2016	FRANCE	*USH2A*	p.(Leu1317ter)	p.(Glu767Serfs*21)
TR_IN-273	FR02U135262	Bonnet et al, 2016	FRANCE	*USH2A*	p.(Glu767Serfs*21)	p.(Thr4809Ile)
TR_IN-274	FR02U3953202	Bonnet et al, 2016	FRANCE	*USH2A*	p.(Val218Glu)	p.(Gly660arg)
TR_IN-275	FR02U2651202	Bonnet et al, 2016	FRANCE	*USH2A*	p.(His308Serfs*16)	p.(Asn346His)
TR_IN-276	FR02U2934662+	Bonnet et al, 2016	FRANCE	*USH2A*	p.(Gly3264Valfs*37)	p.(Arg5143His)
TR_IN-281	FR02U2934662+	Bonnet et al, 2016	FRANCE	*USH2A*	p.(Gly3264Valfs*37)	p.(Arg5143His)
TR_IN-282	FR02U1815092	Bonnet et al, 2016	FRANCE	*USH2A*	c.12067-2A>G	p.(Pro4735Arg)
TR_IN-285	FR02U3342772	Bonnet et al, 2016	FRANCE	*USH2A*	p.(Gly1841Glu)	p.(Gly268Arg)
TR_IN-296	FR02U3137132	Bonnet et al, 2016	FRANCE	*USH2A*	p.(Glu2288ter)	p.(Thr4999Ile)
TR_IN-306	FR02U75U1540	Bonnet et al, 2016	FRANCE	*USH2A*	c.(651+1_652-1)_(784+1_785-1)del	p.(Ser3005Thr)
TR_IN-316	FR02U92U741	Bonnet et al, 2016	FRANCE	*USH2A*	p.(Pro3116Hisfs*13)	p.(Thr4337Met)
TR_IN-317	FR02U05242	Bonnet et al, 2016	FRANCE	*USH2A*	p.(Ile1557Leufs*18)	c.3317-1G>A
TR_IN-320	FR02U2730432	Bonnet et al, 2016	FRANCE	*USH2A*	p.(Ser1247Lysfs*4)	c.9371+1G>T
TR_IN-321	FR02U2327632	Bonnet et al, 2016	FRANCE	*USH2A*	p.(Glu767Serfs*21)	c.3317-2A>G
TR_IN-322	FR02U3750672	Bonnet et al, 2016	FRANCE	*USH2A*	c.13811+2T>G	p.(Pro3272Leu)
TR_IN-324	FR02U2830812	Bonnet et al, 2016	FRANCE	*USH2A*	p.(Gly4095Ser)	p.(Cys759Phe)
TR_IN-326	FR02U76U1651	Bonnet et al, 2016	FRANCE	*USH2A*	p.(Gln3959Asnfs*53)	p.(Ala3660fsVal*14)
TR_IN-327	FR02U77U1680	Bonnet et al, 2016	FRANCE	*USH2A*	p.(Lys811ter)	c.7595-2144A>G
TR_IN-328	FR02U85U2531	Bonnet et al, 2016	FRANCE	*USH2A*	p.(Leu4840Pro)	p.(Leu4840Pro)

TR_IN-330	FR02U82U2210	Bonnet et al, 2016	FRANCE	*USH2A*	p.(Asn4079Trpfs*19)	p.(Glu511Lys)
TR_IN-331	FR02U72S57220	Bonnet et al, 2016	FRANCE	*USH2A*	p.(Trp3955ter)	p.(Pro1843Leu)
TR_IN-332	FR02U3646012	Bonnet et al, 2016	FRANCE	*USH2A*	p.(Cys759Phe)	p.(Leu4282Pro)
TR_IN-336	FR02U0163292	Bonnet et al, 2016	FRANCE	*USH2A*	p.(Glu767Serfs*21)	p.(Glu767Serfs*21)
TR_IN-344	FR02U5163062	Bonnet et al, 2016	FRANCE	*USH2A*	p.(Glu767Serfs*21)	p.(Thr4337Met)
TR_IN-348	FR02U3443182	Bonnet et al, 2016	FRANCE	*USH2A*	p.(Glu767Serfs*21)	p.(Ala1711Serfs*6)
TR_IN-349	FR02U71S27881	Bonnet et al, 2016	FRANCE	*USH2A*	p.(Glu767Serfs*21)	c.(6163+1_6164-1)_(6325+1_6326-1)del
TR_IN-350	FR02U5061812	Bonnet et al, 2016	FRANCE	*USH2A*	p.(Thr4337Met)	p.(Cys717Gly)
TR_IN-351	FR02U91U720	Bonnet et al, 2016	FRANCE	*USH2A*	p.(Gln4635ter)	p.(Ile4059Asnfs*40)
TR_IN-357	FR02U3241292	Bonnet et al, 2016	FRANCE	*USH2A*	p.(Trp3955ter)	p.(Arg2509Glyfs*19)
TR_IN-359	FR02U081112	Bonnet et al, 2016	FRANCE	*USH2A*	c.785-6636_1840+208del	c.785-6636_1840+208del
TR_IN-365	FR02U93U920	Bonnet et al, 2016	FRANCE	*USH2A*	p.(Val2714ter)	c.7595-2144A>G
TR_IN-366	FR02U81U2181	Bonnet et al, 2016	FRANCE	*USH2A*	p.(Pro3116Hisfs*13)	p.(Pro3116Hisfs*13)
TR_IN-367	FR02U94U991	Bonnet et al, 2016	FRANCE	*USH2A*	p.(Glu767Serfs*21)	p.(Ser3856Valfs*28)
TR_IN-368	FR01U03A1430	Bonnet et al, 2016	FRANCE	*USH2A*	p.(Leu4282Pro)	p.(Leu4282Pro)
TR_IN-372	FR02U83U2220	Bonnet et al, 2016	FRANCE	*USH2A*	p.(Glu767Serfs*21)	p.(Leu2319Argfs*7)
TR_IN-373	FR02U3036172	Bonnet et al, 2016	FRANCE	*USH2A*	c.11548+2T>G	p.(Glu767Serfs*21)
TR_IN-377	FR02U1915842	Bonnet et al, 2016	FRANCE	*USH2A*	p.(Glu767Serfs*21)	p.(Val218Glu)
TR_IN-401	FR02U2429002	Bonnet et al, 2016	FRANCE	*USH2A*	p.(Glu767Serfs*21)	p.(Gly4095Ser)
TR_IN-402	FR02U3545182	Bonnet et al, 2016	FRANCE	*USH2A*	p.(Arg1653ter)	p.(Cys5122arg)
TR_UE-136	SLO01U0411442	Bonnet et al, 2016	SLOVENIA	*USH2A*	p.(Trp3955ter)	p.(Trp3955ter)
TR_UE-140	SLO01U1411072	Bonnet et al, 2016	SLOVENIA	*USH2A*	c.4627+25435_4987+660del	c.4627+25435_4987+660del
TR_UE-145	SLO01U2811582	Bonnet et al, 2016	SLOVENIA	*USH2A*	c.(784+1_785-1)_(848+1_849-1)del	p.(Gly4857Ala)
TR_UE-152	SLO01U1110012	Bonnet et al, 2016	SLOVENIA	*USH2A*	p.(Cys870ter)	p.(Trp3955ter)
TR_UE-171	SLO01U1211052	Bonnet et al, 2016	SLOVENIA	*USH2A*	p.(Trp3955ter)	p.(Gly1645ter)
TR_UE-172	SLO01U2111392	Bonnet et al, 2016	SLOVENIA	*USH2A*	p.(Trp3955ter)	p.(Gly1645ter)
TR_UE-173	SLO01U1311062	Bonnet et al, 2016	SLOVENIA	*USH2A*	p.(Trp3955ter)	p.(Phe78Val)
TR_UE-174	SLO01U15111	Bonnet et al, 2016	SLOVENIA	*USH2A*	p.(Cys870ter)	p.(Cys870ter)

	02					
TR_UE-225	SLO01U10STG 02062	Bonnet et al, 2016	SLOVENIA	*USH2A*	p.(Trp3955ter)	p.(Trp3955ter)
TR_UE-227	SLO01U16111 32	Bonnet et al, 2016	SLOVENIA	*USH2A*	p.(Trp3955ter)	p.(Thr352Ile)
TR_UE-228	SLO01U01111 42	Bonnet et al, 2016	SLOVENIA	*USH2A*	p.(Trp3955ter)	p.(Trp3955ter)
TR_UE-233	SLO01U17111 92	Bonnet et al, 2016	SLOVENIA	*USH2A*	p.(Trp3955ter)	p.(Thr3676Ile)
TR_UE-234	SLO01U18112 12	Bonnet et al, 2016	SLOVENIA	*USH2A*	p.(Trp3955ter)	p.(Gly1645ter)
TR_UE-235	N/A		SLOVENIA	*USH2A*	p.(Trp3955ter)	p.(Cys870ter)
TR_UE-289	SLO01U22114 22	Bonnet et al, 2016	SLOVENIA	*USH2A*	p.(Trp3955ter)	p.(Arg303His)
TR_UE-292	SLO01U20113 62	Bonnet et al, 2016	SLOVENIA	*USH2A*	p.(Trp3955ter)	p.(Arg2914ter)
TR_UE-381	SLO01U02113 22	Bonnet et al, 2016	SLOVENIA	*USH2A*	p.(Trp3955ter)	p.(Trp3955ter)
TR_UE-382	SLO01U03113 32	Bonnet et al, 2016	SLOVENIA	*USH2A*	p.(Trp3955ter)	p.(Trp3955ter)
TR_UE-384	SLO01U19113 52	Bonnet et al, 2016	SLOVENIA	*USH2A*	p.(Trp3955ter)	p.(Gly4763arg)
TR_UE-385	N/A		SLOVENIA	*USH2A*	p.(Trp3955ter)	p.(Trp3955ter)
TR_UE-386	SLO01U23114 32	Bonnet et al, 2016	SLOVENIA	*USH2A*	p.(Trp3955ter)	p.(Gln3292ter)
TR_UE-387	SLO01U24114 52	Bonnet et al, 2016	SLOVENIA	*USH2A*	p.(Trp3955ter)	p.(Arg303His)
TR_UE-388	N/A		SLOVENIA	*USH2A*	p.(Trp3955ter)	p.(Phe78Val)
TR_UE-389	SLO01U25115 42	Bonnet et al, 2016	SLOVENIA	*USH2A*	p.(Cys870ter)	p.(Thr4315Pro)
TR_UE-391	SLO01U26115 62	Bonnet et al, 2016	SLOVENIA	*USH2A*	p.(Trp3955ter)	p.(Cys870ter)
TR_UE-392	SLO01U27115 72	Bonnet et al, 2016	SLOVENIA	*USH2A*	p.(Trp3955ter)	p.(Arg1549ter)
TR_UE-395	SLO01U29121 42	Bonnet et al, 2016	SLOVENIA	*USH2A*	p.(Trp3955ter)	p.(Arg626ter)
TR_UE-396	SLO01U30122 52	Bonnet et al, 2016	SLOVENIA	*USH2A*	p.(Trp3955ter)	p.(Arg626ter)
TR_UE-397	SLO01U31122 72	Bonnet et al, 2016	SLOVENIA	*USH2A*	p.(Cys870ter)	p.(Cys870ter)
TR_UE-399	N/A		SLOVENIA	*USH2A*	p.(Trp3955ter)	p.(Arg626ter)
TR_UE-403	SLO01U05123	Bonnet et al, 2016	SLOVENIA	*USH2A*	p.(Trp3955ter)	p.(Trp3955ter)

	62					
TR_UE-404	SLO01U0612372	Bonnet et al, 2016	SLOVENIA	*USH2A*	p.(Trp3955ter)	p.(Trp3955ter)
TR_UE-405	N/A		SLOVENIA	*USH2A*	p.(Trp3955ter)	p.(Trp3955ter)
TR_UE-406	N/A		SLOVENIA	*USH2A*	p.(Trp3955ter)	p.(Trp3955ter)
TR_UE-407	N/A		SLOVENIA	*USH2A*	p.(Trp3955ter)	p.(Trp3955ter)
TR_UE-408	SLO01U3512672	Bonnet et al, 2016	SLOVENIA	*USH2A*	p.(Cys870ter)	p.(Cys870ter)
TR_UE-409	SLO01U3212512	Bonnet et al, 2016	SLOVENIA	*USH2A*	p.(Arg626ter)	p.(Phe78Val)
TR_UE-411	SLO01U0712562	Bonnet et al, 2016	SLOVENIA	*USH2A*	p.(Trp3955ter)	p.(Trp3955ter)
TR_UE-412	SLO01U3312582	Bonnet et al, 2016	SLOVENIA	*USH2A*	p.(Trp3955ter)	p.(Pro1243Leufs*6)
TR_UE-413	SLO01U3612722	Bonnet et al, 2016	SLOVENIA	*USH2A*	p.(Cys870ter)	p.(Arg303His)
TR_UE-414	N/A		SLOVENIA	*USH2A*	p.(Trp3955ter)	p.(Pro1243Leufs*6)
TR_UE-415	SLO01U3412612	Bonnet et al, 2016	SLOVENIA	*USH2A*	p.(Trp3955ter)	p.(Pro2241His)
TR_UE-416	N/A		SLOVENIA	*USH2A*	p.(Trp3955ter)	p.(Trp3955ter)
TR_EK-047	DE03G03480+	Bonnet et al, 2016	GERMANY	*ADGRV1*	p.(Gln753Leufs*8)	p.(Lys1786Ilefs*8)
TR_EK-048	DE03G03480+	Bonnet et al, 2016	GERMANY	*ADGRV1*	p.(Gln753Leufs*8)	p.(Lys1786Ilefs*8)
TR_EK-055	DE03G04551	Bonnet et al, 2016	GERMANY	*ADGRV1*	p.(Tyr536ter)	p.(Tyr536ter)
TR_EK-061	DE03G05611	Bonnet et al, 2016	GERMANY	*ADGRV1*	p.(Phe5328Serfs*41)	p.(Glu2669Lysfs*4)
TR_EK-087	DE03G06870	Bonnet et al, 2016	GERMANY	*ADGRV1*	p.(Trp3486ter)	p.(Glu6295Alafs*31)
TR_EK-121	DE03G011211+	Bonnet et al, 2016	GERMANY	*ADGRV1*	p.(Arg3293ter)	p.(Ser1358Pro)
TR_EK-126	DE03G011211+	Bonnet et al, 2016	GERMANY	*ADGRV1*	p.(Arg3293ter)	p.(Ser1358Pro)
TR_EK-175	DE03G021752	Bonnet et al, 2016	GERMANY	*ADGRV1*	p.(Gly2045ter)	c.10054-1G>T
TR_EK-260	DE03G072602	Bonnet et al, 2016	GERMANY	*ADGRV1*	p.(Arg920ter)	c.(4752+1_4753-1)_(16196+1_16197-1)del
TR_FT-205	IT02G012020	Bonnet et al, 2016	ITALY	*ADGRV1*	p.(Leu568Cysfs*8)	p.(Cys5970Ser)
TR_IN-158	P0473	Bonnet et al, 2011	FRANCE	*ADGRV1*	p.(Pro522Serfs*8)	p.(Met5890Valfs*10)

TR_IN-166	FR02G028272	Bonnet et al, 2016	FRANCE	*ADGRV1*	p.(Arg493ter)	c.1509+3A>G
TR_IN-167	FR02G0315122	Bonnet et al, 2016	FRANCE	*ADGRV1*	p.(Gln5626ter)	p.(Gln5626ter)
TR_IN-168	FR02G0746112	Bonnet et al, 2016	FRANCE	*ADGRV1*	p.(Ser4048ter)	p.(Asp3992Asn)
TR_IN-280	FR02G0534762	Bonnet et al, 2016	FRANCE	*ADGRV1*	p.(Gln3490ter)	p.(Gln3490ter)
TR_IN-303	FR02G0636522	Bonnet et al, 2016	FRANCE	*ADGRV1*	p.(Ala2032Argfs*27)	c.9184+3A>G
TR_IN-340	FR02G0850662	Bonnet et al, 2016	FRANCE	*ADGRV1*	p.(Ser4441Leufs*9)	p.(Val5647Glyfs*7)
TR_IN-356	FR02G011822	Bonnet et al, 2016	FRANCE	*ADGRV1*	p.(Gly2329Argfs*17)	c.9906+1G>A

The data of majority of the patients has been previously reported, as a part of genetic report on Treatrush study - see references. + symbol denotes siblings. Phenotypes of TR_EK-047 and TR_EK-048 have been described previously (Ebermann et al, 2009).
